# Immunomodulatory Properties of Mesenchymal Stem Cells and Their Extracellular Vesicles: Potential Clinical Applications in Autoimmune Diseases

**DOI:** 10.7759/cureus.117095

**Published:** 2026-09-28

**Authors:** Eduardo Ciarrochi, Juan Bautista Ciarrochi

**Affiliations:** 1 Regenerative Medicine, Clínica de Cirugía Especializada, Buenos Aires, ARG

**Keywords:** autoimmune inflammatory diseases, extracellular vesicles, inflammatory diseases, mesenchymal stem cells (mscs), stem cell applications including immunomodulation

## Abstract

Mesenchymal stem cells (MSCs)/mesenchymal stromal cells and their extracellular vesicles (EVs), particularly exosomes, have emerged as therapeutic candidates in regenerative medicine due to their multipotency, low immunogenicity, and extensive immunomodulatory properties. This narrative review provides a narrative synthesis of MSC- and MSC-EV-mediated immunomodulation and its translational potential across four major autoimmune diseases: multiple sclerosis (MS), type 1 diabetes mellitus (T1D), systemic lupus erythematosus (SLE), and rheumatoid arthritis (RA). Mechanistically, MSCs regulate both innate and adaptive immune cell populations, modulate pro- and anti-inflammatory cytokine secretion, downregulate pro-inflammatory Th1/Th17 responses, and promote regulatory phenotypes, including regulatory T cells, regulatory B cells, and M2-polarized macrophages. Preclinical and clinical investigations suggest therapeutic potential across these diseases; however, much of the clinical evidence derives from small, early-phase, and frequently uncontrolled studies with limited follow-up, and safety outcomes vary according to MSC source, manufacturing process, dose, route of administration, and clinical indication. In these studies, the absence of serious adverse events does not constitute definitive evidence of long-term safety. Clinical studies have reported reductions in disease activity scores and inflammatory biomarkers, as well as improvements in disease-specific functional outcomes; however, these findings should be regarded as preliminary evidence of therapeutic potential rather than established efficacy across autoimmune diseases. Nonetheless, clinical translation remains hindered by manufacturing heterogeneity, variability in dosing, routes of administration, and treatment protocols, as well as challenges related to allogeneic immunogenicity and MSC-EV standardization. Overcoming these barriers will require standardized manufacturing and characterization protocols, alongside further investigation into the integration of MSC- and MSC-EV-based therapies within multimodal, personalized, and patient-tailored treatment frameworks.

## Introduction and background

Therapeutic interest in mesenchymal stem cells (MSCs)/mesenchymal stromal cells has grown considerably over the past decade, yet their translation into routine clinical practice still faces substantial challenges [1]. Among the different stem cell populations, MSCs have gained considerable attention because of their biological properties and their role in regenerative medicine [2]. MSCs are multipotent cells characterized by their self-renewal capacity, relatively low immunogenicity in their resting state, homing ability, and immunomodulatory properties, although their immunogenicity can be enhanced under inflammatory conditions or in allogeneic settings. Among these biological features, immunomodulation has emerged as one of the principal mechanisms underlying their therapeutic potential, allowing MSCs to regulate both innate and adaptive immune responses. Consequently, these cells have emerged as therapeutic candidates for the treatment of immune-mediated disorders, particularly autoimmune and inflammatory diseases [3,4]. A substantial part of this activity is exerted through secreted products rather than through direct cell-to-cell contact, including extracellular vesicles (EVs) that transfer proteins, lipids, and nucleic acids to recipient cells and are increasingly explored as cell-free therapeutic alternatives [5].

Throughout this review, the MSC abbreviation is used in line with current nomenclature discussions, encompassing both "mesenchymal stem cells" and "mesenchymal stromal cells," given that the therapeutic effects of these cells are now widely attributed primarily to paracrine and immunomodulatory mechanisms rather than to stemness or durable engraftment [6,7]. The abbreviation is retained consistently throughout the manuscript in this stromal/paracrine sense.

Autoimmune diseases are a heterogeneous group of conditions caused by the immune system's response to self-antigens, resulting in self-tissue damage or dysfunction [8]. The age-standardized prevalence of autoimmune diseases has nearly doubled between 1990 and 2021 [9]. However, prevalence estimates vary considerably across diseases and populations, with population-based studies reporting a prevalence of approximately 3-5% in the general population [10,11]. Current therapeutic strategies include corticosteroids, non-steroidal anti-inflammatory drugs, biologic agents, resolvins, and other immunomodulatory medications [12]. Although these treatments have substantially improved disease management, long-term therapy may be associated with renal, gastrointestinal, and cardiovascular toxicity. Consequently, there remains a need for therapeutic strategies capable of controlling inflammation while minimizing systemic toxicity and providing more selective immunomodulatory effects [13]. These considerations, together with the immunomodulatory properties of MSCs and the evidence generated by preclinical and clinical studies, have prompted the investigation of MSC-based therapy in autoimmune and inflammatory diseases [14].

Despite the progress summarized above, a clinically relevant knowledge gap persists. Although the immunomodulatory properties of MSCs and MSC-EVs have been extensively characterized in experimental models, the evidence supporting their clinical application remains heterogeneous and, in some cases, inconsistent, with variable outcomes largely attributed to differences in cell sources, manufacturing processes, and routes of administration [15]. Furthermore, MSC-EV-based therapies remain predominantly confined to the preclinical stage, with unresolved challenges related to large-scale production, isolation, and characterization [16]. Importantly, the available evidence is fragmented across individual diseases and therapeutic modalities, and an integrated synthesis that connects the mechanistic basis of MSC- and MSC-EV-mediated immunomodulation with the current status of clinical translation in autoimmune diseases is still lacking. This review was conceived to address this gap. 

The aim of the present narrative review is to provide a synthesis of the immunomodulatory properties of MSCs and their clinical implications for the treatment of autoimmune and inflammatory diseases, from a translational perspective that integrates mechanistic understanding with the available preclinical and clinical evidence. Rather than providing a systematic appraisal of trial data, this review offers a mechanism-informed overview of how MSC-mediated immunomodulation has been - and could be - translated into therapeutic strategies. Given the marked heterogeneity of the included studies in terms of study populations, MSC sources, manufacturing methods, dosing regimens, routes of administration, and outcome measures, no formal meta-analysis or statistical pooling was performed; findings are therefore presented descriptively. The manuscript first reviews the biological characteristics of MSCs and their secretome, then examines the molecular and cellular mechanisms of MSC- and MSC-EV-mediated immunomodulation, and finally appraises the preclinical and clinical evidence across four representative diseases (multiple sclerosis (MS), type 1 diabetes mellitus (T1D), systemic lupus erythematosus (SLE), and rheumatoid arthritis (RA)), together with the current limitations, challenges, and future perspectives of MSC-based therapies.

Review design and scope

This article is a narrative review, and its scope and methodological identity are defined accordingly. The principal questions addressed are which immunomodulatory mechanisms underlie the therapeutic effects of MSCs and their EVs, and how these mechanisms have been - and could be - translated into clinical application in autoimmune and inflammatory diseases. The emphasis of the review is mechanistic and translational rather than epidemiological or trial-methodological in isolation, and the four diseases discussed - MS, T1D, SLE, and RA - were selected because, together, they represent distinct target tissues, distinct pathogenic mechanisms, and the full range of clinical evidence currently available in this setting, from randomized controlled trials to open-label cohorts and individual clinical observations. In accordance with the narrative design of this article, study selection was not systematic. Publications were identified and selected on the basis of thematic relevance and the strength of the available evidence, with priority given to the most representative studies for each disease. No formal risk-of-bias assessment, predefined protocol, or quantitative synthesis was performed, and the available evidence is presented descriptively, with its strengths and limitations explicitly noted where clinical outcomes are discussed. This review therefore does not constitute a systematic or exhaustive synthesis of all available evidence, and it does not claim to follow systematic review methodology.

Literature identification and selection

A literature search was conducted in PubMed and Google Scholar, without date restriction, covering publications from 1966 to August 2026 (last search conducted in September 2026), using combinations of the terms “mesenchymal stem cells,” “mesenchymal stromal cells,” “extracellular vesicles,” “exosomes,” “immunomodulation,” “autoimmune diseases,” “multiple sclerosis,” “type 1 diabetes mellitus,” “systemic lupus erythematosus,” and “rheumatoid arthritis.” Consistent with the narrative design of this review, the search is reported descriptively, and no single reproducible Boolean search string, record count, or duplicate-screening procedure was used. Foundational studies on MSC biology and immunomodulatory properties were identified through broader searches of the MSC literature also performed without date restriction, which accounts for the historical range of the publications cited. Preclinical studies, clinical trials, case reports, systematic reviews, and meta-analyses published in English were considered. Recent systematic reviews, randomized trials, and key consensus/guideline documents were specifically sought and incorporated. Where findings were conflicting or heterogeneous, the discrepancies were acknowledged and discussed in the corresponding sections, with the strengths and limitations of the available evidence taken into account. The restriction to English-language publications may have excluded relevant non-English studies.

## Review

Biological characteristics of MSCs

MSCs are multipotent progenitor cells with broad anti-inflammatory and immunomodulatory properties, and they remain among the most extensively studied adult stem cell populations in clinical testing [17]. Friedenstein et al. reported and identified clonogenic progenitor cells capable of generating fibroblast colonies and differentiating into other mesodermal cell lineages, laying the foundations for the subsequent characterization of MSCs [18,19]. The first clinical trial using autologous MSCs was conducted by Lazarus et al. in 1995. The procedure was performed in 15 patients, with no serious adverse events or significant toxicity reported following the intravenous infusion of culture-expanded MSCs [20].

MSCs are multipotent cells capable of differentiating into several mesodermal lineages, including osteoblasts, chondrocytes, and adipocytes, while differentiation toward ectodermal and endodermal lineages has also been reported under specific experimental conditions. Their ability to modulate immune responses and promote tissue regeneration distinguishes MSCs from other cell populations and supports their therapeutic potential [21]. Due to their immunomodulatory, paracrine, and regenerative properties, MSCs have been extensively investigated for potential clinical applications [22]. Although the biological characteristics of MSCs vary according to their tissue of origin, they generally share self-renewal ability, immune regulatory properties, clonogenic capacity, and similar morphological features [23]. To establish standardized criteria for their identification, the International Society for Cellular Therapy (ISCT) proposed the following criteria: 1) Adherence to plastic in standard conditions; 2) Phenotype positive for CD105, CD73, CD90 and negative for CD45, CD34, CD14, CD11b, CD79α, CD19, and human leukocyte antigen (HLA)-DR; 3) In vitro differentiation into osteoblasts, adipocytes, and chondroblasts (demonstrated by staining of in vitro cell culture) [24]. However, the 2006 minimal criteria are now considered insufficient to determine the clinical potency of MSCs, and the ISCT has promoted the development of mechanism-of-action-based functional potency assays as a criterion for product release, as discussed in its recent workshops on the use of MSCs in autoimmune diseases [25].

In this context, the ISCT has proposed that immune functional potency testing for advanced-phase trials should rely on a matrix of analytic methods, including quantitative RNA analysis, flow cytometry of functionally relevant surface markers, and protein-based assays of the MSC secretome [26]. Nevertheless, potency assays and biomarkers for MSC-based advanced therapy medicinal products remain an active area of methodological development [27].

MSCs can be isolated from a wide variety of adult tissues, including bone marrow, adipose tissue, peripheral blood, lungs, and heart [28]. MSCs have also been associated with perivascular cells, particularly pericytes, supporting the presence of MSC populations in perivascular niches throughout the body [29]. In addition, perinatal tissues represent important sources of MSCs, including the umbilical cord, umbilical cord blood, and Wharton's jelly [30]. Among the multiple available sources, this review focuses primarily on adipose tissue-derived mesenchymal stem cells (ADSCs), bone marrow-derived mesenchymal stem cells (BM-MSCs), and umbilical cord-derived mesenchymal stem cells (UC-MSCs).

ADSCs represent an attractive source of MSCs because sufficient amounts of adipose tissue can be obtained through relatively simple and minimally invasive procedures [31]. In addition, adipose tissue generally provides a higher yield of MSCs than bone marrow, potentially facilitating the procurement of larger numbers of cells for expansion and therapeutic applications [32]. Interest in ADSCs has progressively increased because of their anti-apoptotic, anti-inflammatory, immunomodulatory, and pro-angiogenic properties, as well as the biological activity of their secreted products [33].

BM-MSCs are a population of multipotent stromal cells present within the bone marrow and characterized by relatively low immunogenicity [34]. These cells can contribute to regenerative processes in different tissues through several mechanisms, including the secretion of growth factors, differentiation into specialized cell types, and modulation of immune responses [35]. However, the procurement of BM-MSCs generally requires an invasive and potentially painful procedure, and the number of MSCs obtained from bone marrow aspirates may be relatively limited, representing practical disadvantages compared with other tissue sources [36].

UC-MSCs are found in several compartments of the umbilical cord, particularly within Wharton's jelly and perivascular regions [37]. One of their distinctive biological characteristics is their high proliferative capacity, reflected by a shorter population doubling time compared with that of several other MSC populations [38]. UC-MSCs also exhibit relevant immunomodulatory properties through both direct interactions with immune cells and the secretion of paracrine factors, characteristics that have contributed to growing interest in their potential therapeutic application across a variety of diseases [39].

MSCs can be obtained from either autologous sources (in which cells are derived from the same patient who will receive them) or allogeneic sources (in which cells are obtained from a donor). Both approaches present distinct advantages and limitations. Autologous MSCs minimize the risk of immune rejection and generally do not require concomitant immunosuppressive therapy; however, their clinical application may be limited by the time required for cell isolation and expansion, reduced immediate availability, and the potential influence of patient-specific characteristics or underlying disease on cell quality and functionality. In contrast, allogeneic MSCs enable donor selection, can be manufactured and stored in advance for immediate “off-the-shelf” availability, and generally exhibit relatively low immunogenicity [40]. Nevertheless, allogeneic cell-based therapeutic approaches may involve immunological and treatment-related risks [41]. In the specific setting of allogeneic MSC therapy, donor-related variability, host immune recognition, limited persistence after administration, and potential alloimmune responses represent additional considerations that may influence therapeutic efficacy and safety [40]. This concern is particularly relevant for the treatment of chronic autoimmune diseases, in which repeated infusions are commonly required. Although allogeneic MSCs were initially considered immune privileged due to their low expression of major histocompatibility complex (MHC) and co-stimulatory molecules, they can be recognized by the host immune system and have been shown to elicit anti-donor immune responses involving dendritic cells (DCs), T cells, and B-cell-mediated alloantibodies [42]. In clinical studies, donor-specific antibodies developed in a mean of 11.51% of patients treated with allogeneic MSCs; however, neither the production of donor-specific antibodies after treatment nor the presence of donor-specific antibodies at baseline (presensitization) correlated with the safety or tolerability of the therapy, and the generation of alloantibodies might not have clinical relevance [43].

 As discussed throughout this narrative review, a substantial proportion of the biological and therapeutic effects attributed to MSCs may be mediated through the secretion of paracrine factors [44]. Among these secreted components, MSC-derived exosomes (MSC-Exos) have attracted considerable attention because they transport biologically active cargo, including proteins, nucleic acids, lipids, and microRNAs (miRNAs), thereby contributing to intercellular communication and potentially mediating anti-inflammatory effects within inflammatory microenvironments [45]. Given this, the following section examines the MSC secretome and its major components, with particular emphasis on EVs and exosomes.

The MSC secretome and its components

The cell secretome comprises a complex repertoire of biologically active products released by cells into the extracellular environment, including soluble factors, such as growth factors, cytokines, chemokines, and hormones, as well as EVs carrying a variety of bioactive molecules [46]. Among the soluble components, the MSC secretome contains several cytokines, including IL-10, CCL2, IL-6, and IL-7, which contribute to the paracrine and immunomodulatory activity of MSCs. In addition, MSCs secrete a broad range of growth factors and anti-inflammatory mediators that further support the biological activity of the secretome [47,48]. Regarding EVs, according to the Minimal Information for Studies of Extracellular Vesicles 2023 (MISEV2023) guidelines, EVs can be classified based on their physical characteristics, such as size, into "small EVs" and "medium/large EVs," with size ranges of <100 nm or <200 nm for small EVs and >200 nm for medium/large EVs, respectively [49].

 Exosomes are nanoscale EVs (small EVs) enclosed by a lipid bilayer and naturally released by cells. They function as key mediators of intercellular communication through the transfer of proteins, nucleic acids, lipids, and other bioactive molecules to recipient cells [50]. Through the delivery of this molecular cargo, exosomes can transmit biological signals to recipient cells throughout the body, playing an important role in cell signaling and the modulation of cellular functions [51]. Among their bioactive components, MSC-Exos are particularly enriched in miRNAs and contain a complex molecular composition including lipids, enzymes, nucleic acids, proteins, and pro- and anti-inflammatory cytokines, which contributes to protecting miRNAs from degradation by ribonucleases in body fluids. Following the uptake of MSC-Exos by recipient cells, miRNAs are released and can target and silence specific mRNAs, thereby modulating gene expression and influencing the physiological functions of recipient cells [52]. MSC-Exos and their miRNA cargo have been investigated for their therapeutic potential in several preclinical models, including acute kidney injury, hepatic injury, and myocardial ischemia-reperfusion injury [53-55]. Furthermore, MSC-Exos have been associated with the regulation and attenuation of inflammatory responses through the transfer of bioactive cargo (including miRNAs) to recipient cells, supporting their potential contribution to the immunomodulatory effects attributed to MSCs [56].

EVs and exosomes secreted by MSCs can exert paracrine effects on various immune cell populations, contributing to the modulation of both innate and adaptive immune responses [57]. MSC-Exos retain many of the potential therapeutic properties attributed to their cells of origin, including anti-inflammatory, immunomodulatory, and regenerative effects [58]. Consequently, MSC-Exos and MSC-EVs have attracted considerable interest in biomedical research because of their ability to influence tissue responses to injury and disease, representing a promising alternative for the development of cell-free regenerative medicine strategies [44].

Immunomodulatory properties of MSCs and MSC-EVs

Several biological characteristics appear to contribute to the immunomodulatory and immunoregulatory properties of MSCs. MSCs are considered hypoimmunogenic due to their low expression of MHC/HLA class I, negligible expression of MHC/HLA class II, and low expression of co-stimulatory molecules, including CD40, CD40 ligand, CD80, and CD86 [59,60]. MSCs also secrete several soluble factors, such as indoleamine 2,3-dioxygenase (IDO), nitric oxide, and IL-6, which contribute through paracrine mechanisms to the modulation and inhibition of immune responses [61-63]. MSCs exert extensive immunomodulatory effects on both innate and adaptive immune responses [64]. In addition, MSC-EVs can modulate these responses by suppressing pro-inflammatory cytokines and influencing the activity of macrophages and T cells [65].

The immunomodulatory properties of MSCs are not entirely constitutive but are strongly influenced by the surrounding inflammatory microenvironment. The crosstalk between MSCs and the inflammatory microenvironment is bidirectional, and the characteristics of the inflammatory milieu can drive MSCs toward either immunosuppressive or immune-enhancing functions [66]. A study conducted by Li et al. demonstrated that pro-inflammatory cytokines play a crucial role in determining the immunomodulatory functions of MSCs. The co-presence of interferon-γ (IFN-γ) and another pro-inflammatory cytokine, such as tumor necrosis factor-α (TNF-α), was required for MSCs to exert their immunosuppressive effects [67]. Similarly, Ren et al. showed that the immunosuppressive function of MSCs is induced by pro-inflammatory cytokines and requires nitric oxide. Furthermore, pro-inflammatory cytokines induce MSCs to produce soluble factors that are crucial for their immunosuppressive activity [68]. However, this same inflammatory licensing can also raise MSC immunogenicity by increasing MHC expression, so the balance between immunomodulatory activation and immunogenic risk should be considered, particularly in allogeneic settings [69].

The elicitation of the immunosuppressive functions of MSCs requires an inflammatory environment; therefore, understanding the inflammatory milieu present in different pathological conditions may be important for optimizing their immunomodulatory function and therapeutic efficacy [70]. For instance, studies using models of inflammatory and immune-mediated diseases, including graft-versus-host disease (GVHD) and experimental autoimmune encephalomyelitis (EAE), have suggested that when MSCs are administered at the onset of disease, when inflammatory signals are still limited, their therapeutic efficacy may be reduced [71,72].

MSCs possess important immunoregulatory properties, acting on different immune cell populations both directly through cell-to-cell contact and indirectly through paracrine mechanisms mediated by soluble factors and MSC-EVs; accordingly, MSCs interact with T cells, B cells, macrophages, natural killer (NK) cells, and DCs [73]. Given the central role of these immune cell populations in MSC-mediated immunomodulation, their principal interactions with MSCs and MSC-EVs are schematically illustrated below (Figure 1), while the following sections examine their immunomodulatory effects in greater detail.

**Figure 1:**
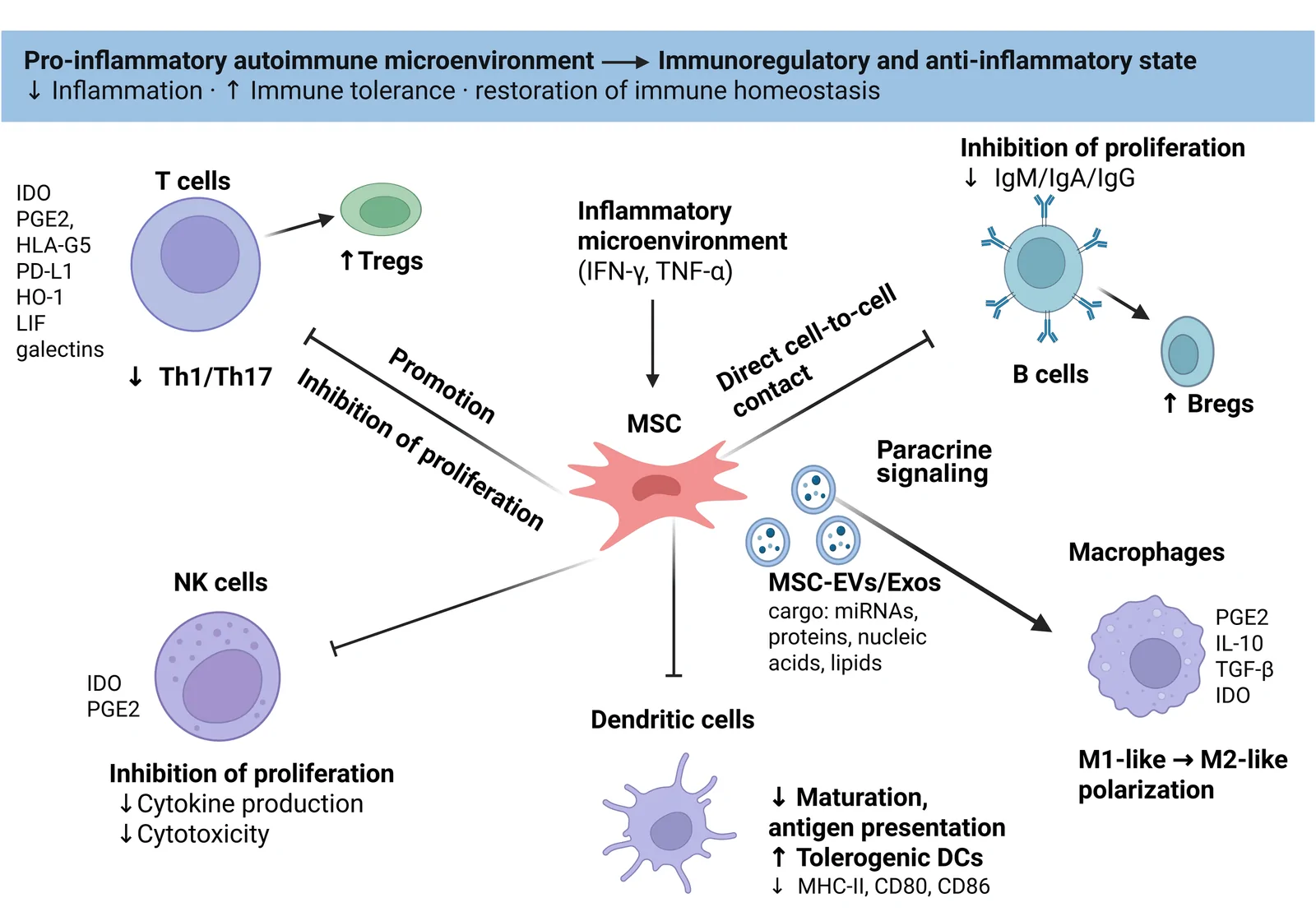
Immunomodulatory mechanisms of MSCs and MSC-EVs Schematic representation of MSC- and MSC-EV-mediated regulation of innate and adaptive immune cell populations. Through direct cell-to-cell contact and paracrine signaling - including the release of soluble mediators, EVs, and exosomes - MSCs modulate effector functions in T cells, B cells, NK cells, macrophages, and DCs. These concerted cellular interactions promote a functional shift from a pro-inflammatory microenvironment toward an immunoregulatory state, contributing to the attenuation of inflammation, enhancement of immune tolerance, and restoration of immune homeostasis. Created in BioRender. Ciarrochi JB (2026) https://BioRender.com/nfp8rps. MSC: Mesenchymal stem cell; MSC-EV: Mesenchymal stem cell-extracellular vesicle; NK: Natural killer; DC: Dendritic cell

Immunomodulatory effects of MSCs and MSC-EVs on T cells

T cells are a key cellular component of the adaptive immune system, playing a fundamental role in maintaining health and preventing different types of diseases. MSCs can modulate T cell functions either indirectly, by acting on other immune cells that subsequently influence T cell responses, or directly, through their effects on T cells [74]. Regarding the indirect pathway, MSCs can maintain macrophages and DCs in an immature state, thereby preventing the activation of effector T cells and promoting the generation of Tregs [75]. Regarding the direct pathway, soluble factors secreted by MSCs can modulate T cell responses; for example, MSC-derived HLA-G5 has been shown to suppress T cell functions and promote the generation of CD4+CD25highFOXP3+ Tregs [76]. 

Although MSCs themselves generally do not secrete the inflammatory cytokines TNF-α and IFN-γ, these cytokines - produced by surrounding immune cells - act as licensing signals that enhance the secretion of immunosuppressive mediators such as prostaglandin E2 (PGE2) and IDO, thereby inhibiting T cell proliferation and modulating the adaptive immune response [77]. Furthermore, MSCs produce immunosuppressive molecules such as heme oxygenase-1 (HO-1), leukemia inhibitory factor (LIF), programmed death-ligand 1 (PD-L1), and galectins, which contribute to the inhibition of T cell differentiation and proliferation [78-82].

MSCs can also influence the balance between pro-inflammatory and regulatory T cell subsets. They can suppress Th17 differentiation through downregulation of RORγt while promoting the Treg phenotype through increased Foxp3 expression, thereby contributing to the regulation of the Th17/Treg balance and attenuation of inflammatory responses [52].

MSC-EVs can reduce T cell activation and enhance Treg cell activity [83]. In a study conducted by Yeganeh et al., MSC-EVs were associated with an increased frequency of CD4+CD25+FOXP3+ T cells, together with upregulation of Foxp3 mRNA expression [84].

Immunomodulatory effects of MSCs and MSC-EVs on B cells

B cells mediate humoral immunity primarily via the synthesis and secretion of antibodies; they also serve as antigen-presenting cells and regulate the function of other immune cells [85].

In a study conducted by Corcione et al., MSCs inhibited B cell proliferation through G0/G1 cell cycle arrest; additionally, IgM, IgA, and IgG production was significantly reduced in B cells co-cultured with MSCs compared with control B cells [86]. Inflammatory stimulation can enhance the inhibitory effects of MSCs on B cells, with IFN-γ playing an important role in this response [87]. MSCs can also induce the generation of CD23+CD43+ regulatory B cells (Bregs), which are capable of suppressing T cell proliferation and pro-inflammatory cytokine production, suggesting a potential therapeutic role in inflammation-related diseases [88].

MSC-EVs can suppress B cell proliferation, with a more pronounced inhibitory effect observed when EVs are derived from inflammatory-primed MSCs [89]. BMSC-Exos can modulate B cell function by regulating the expression of genes involved in B cell maturation and differentiation [90]. 

Immunomodulatory effects of MSCs and MSC-EVs on NK cells

NK cells are key effectors of innate immunity; they become activated upon encountering infected, allogeneic, and tumor cells [91]. MSCs can modulate the NK cell phenotype and inhibit their proliferation, cytokine secretion, and cytotoxic activity against HLA class I-expressing target cells [92]. MSCs can inhibit IL-2-induced NK cell proliferation and effector functions, including cytotoxic activity and cytokine production, with IDO and PGE2 acting as key mediators of MSC-induced NK cell suppression [93].

MSC-EVs can modulate NK cell functions in different inflammatory settings, suppressing their pro-inflammatory and cytotoxic activity [94]. The ADSC secretome restricts IL-2-mediated NK cell effector functions through the upregulation of cytokine-inducible SH2-containing protein (CIS) and dual-specificity phosphatase 4 (DUSP4), thereby supporting its therapeutic potential in inflammatory and autoimmune diseases [95]. 

Immunomodulatory effects of MSCs and MSC-EVs on macrophages and DCs

Macrophages are widely distributed immune cells that reside in tissues throughout the body and constitute an important component of the mononuclear phagocyte system, playing essential roles in immune defense, surveillance, and tissue homeostasis [96]. Given the narrative nature of this review, macrophages will be described according to the simplified M1/M2 classification, which is used here for heuristic purposes despite representing an oversimplification, as current evidence indicates that macrophage activation spans a continuous spectrum of functional states determined by the local microenvironment rather than discrete subsets. M1 macrophages are effector cells characterized by the secretion of pro-inflammatory cytokines, whereas M2 macrophages produce high levels of anti-inflammatory cytokines, including IL-10 and transforming growth factor-β (TGF-β), and play important roles in wound healing and tissue repair [97].

MSCs can promote the shift from pro-inflammatory M1-type macrophages toward an anti-inflammatory M2-type phenotype through IL-10-mediated mechanisms [98]. Injected BM-MSCs interact with macrophages, reprogramming them. MSCs produce PGE2 that acts on macrophages, stimulating IL-10 production, an anti-inflammatory cytokine [99]. A study conducted by Vasandan et al. showed that MSC-secreted PGE2 plays a crucial role in modulating macrophage activation, promoting a shift from the M1-type toward the M2-type phenotype [100]. These IL-10-secreting M2-type macrophages can suppress T cell proliferation and inhibit B cell differentiation [101,102].

MSCs can modulate macrophage function through soluble mediators, including IL-10, TGF-β, IDO, and PGE2 [63]. MSC-EVs play a crucial role in these mechanisms. CCR2-positive MSC-Exos can bind CCL2 and suppress its ability to recruit and activate macrophages [103]. Atorvastatin-pretreated BM-MSC-EVs can promote M2 macrophage polarization through the transfer of miR-139-3p, which suppresses the STAT1 pathway and contributes to cardiac repair following myocardial infarction [104].

DCs are innate immune cells and constitute one of the most effective types of antigen-presenting cells, playing a central role in coordinating various forms of immune responses [105]. MSCs can modulate DC phenotype, differentiation, and migration, while weakening their antigen presentation ability [106]. MSCs can promote the reversion of mature DCs toward an immature phenotype, characterized by downregulation of MHC-II and the costimulatory molecules CD80 and CD86, thereby reducing their capacity to stimulate T cell responses [107].

MSC-EVs play a crucial role in the regulation of DC functions in various inflammatory and immune-mediated diseases [108]. They can be internalized by DCs and promote a tolerogenic DC phenotype, while also inducing a shift toward an anti-inflammatory Treg profile, a finding with potential relevance for autoimmune diseases such as T1D [109]. 

Clinical implications of MSC- and MSC-EV-mediated immunomodulation in autoimmune and inflammatory diseases

Inflammation is a physiological response triggered by the body as a defense mechanism against infectious and injurious agents. Under normal conditions, this process is self-limited and resolves once the infectious or injurious stimulus has ceased [110]. However, despite its physiological role, when inflammation becomes chronic and unresolved, it can lead to tissue damage and perpetuate various pathological processes [111]. Chronic inflammation is associated with several health conditions and contributes to increased morbidity and mortality in diseases such as chronic kidney disease, cardiovascular disease, diabetes mellitus, neurodegenerative disorders, and autoimmune diseases [112].

Clinical studies of MSC-based therapies have generally shown an acceptable short-term safety profile, although most of the available evidence derives from early-phase and frequently uncontrolled studies [113]. However, the absence of serious adverse events in small or early-phase studies should not be interpreted as definitive evidence of long-term safety, and the limited follow-up and methodological heterogeneity of available trials warrant caution. Supported by their immunomodulatory and anti-inflammatory properties and by the acceptable short-term safety profile observed in early-phase trials, MSCs have been administered predominantly in clinical trials for the treatment of autoimmune diseases, particularly in patients who had exhausted other therapeutic options [114]. These therapies have been investigated across a broad spectrum of autoimmune and inflammatory conditions, including Crohn’s disease, GVHD, psoriasis, liver injury, primary Sjögren’s syndrome, T1D, MS, SLE, and RA, among others [57,115].

In the following sections, we will explore in depth the clinical translation of these therapies across four selected diseases - MS, T1D, SLE, and RA - whose potential therapeutic applications are illustrated below (Figure 2). We will analyze their pathophysiology, the therapeutic mechanisms involved, and the available clinical evidence translating the immunomodulatory properties of MSCs into clinical practice.

**Figure 2:**
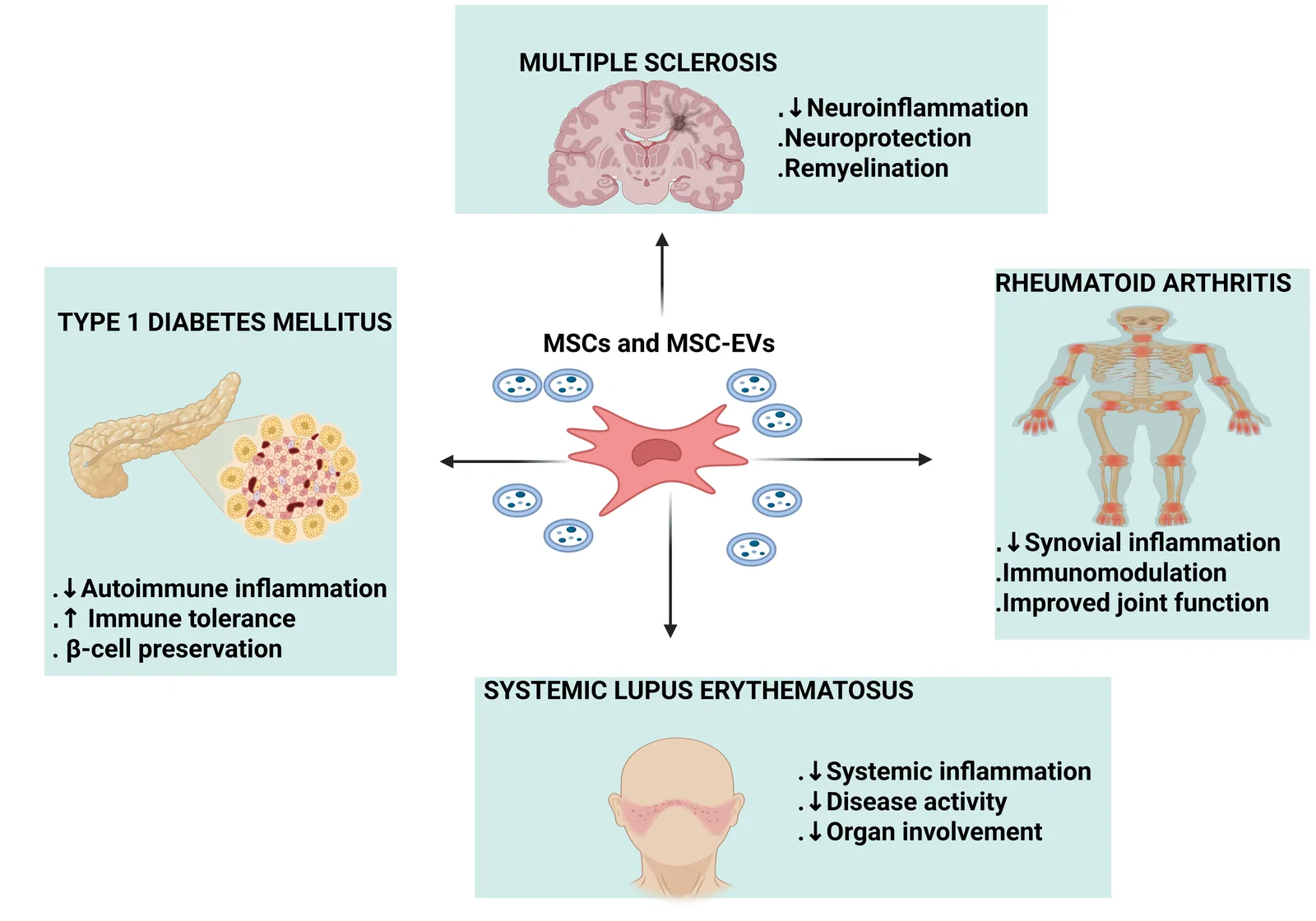
Potential therapeutic applications of MSCs and MSC-EVs in autoimmune diseases The immunomodulatory properties of MSCs and MSC-EVs support their potential therapeutic application in MS, T1D, SLE, and RA. Their reported effects include modulation of inflammatory and autoimmune responses, promotion of immune tolerance, preservation of tissue function, and improvement of disease-related outcomes. Created in BioRender. Ciarrochi JB (2026) https://BioRender.com/cgylyic. MSC: Mesenchymal stem cell; MSC-EV: Mesenchymal stem cell-extracellular vesicle; MS: Multiple sclerosis; T1D: Type 1 diabetes mellitus; SLE: Systemic lupus erythematosus; RA: Rheumatoid arthritis

MS

MS is a chronic autoimmune disease that affects the central nervous system, primarily targeting myelinated axons and causing varying degrees of structural damage to both myelin and axons [116]. Multiple cellular components, including autoreactive CD4+ T cells, B cells, NK cells, macrophages, and microglia, together with soluble mediators such as IFN-γ, IL-6, and IgG1, contribute to MS pathogenesis [117]. Chronic active lesions are characterized by microglial activation, oxidative stress, mitochondrial dysfunction, and iron accumulation, which collectively contribute to progressive neuroaxonal degeneration [118]. Studies have shown that MSCs can modulate MS-associated immune responses by promoting anti-inflammatory Th2 and Treg responses while suppressing pro-inflammatory Th1 and Th17 responses and inflammatory cytokine activity [119]. 

In preclinical models, MSC infusion was effective in decreasing inflammation, preserving axons, promoting oligodendrogenesis, and increasing nerve conduction velocity, improving neurological functions [120]. MSC-released factors also play a crucial role in targeting MS. MSC-derived hepatocyte growth factor (HGF) promotes remyelination and modulates the inflammatory response by reducing pro-inflammatory cytokines such as IFN-γ and IL-17 [121]. Preclinical evidence has also demonstrated the neuroprotective and neuroregenerative potential of MSC-EVs, which protected neurons from apoptotic cell death and promoted remyelination through the activation of oligodendroglial precursors [122]. Exosomes have also been shown to cross the blood-brain barrier, expanding their potential therapeutic relevance in central nervous system disorders [123].

Clinical trials investigating MSC-based therapies for MS have generally reported improvements in neurological outcomes, together with an acceptable safety profile [124]. Several clinical studies have reported favorable outcomes following MSC-based therapies for MS, including fewer disease episodes, reduced annual relapse frequency, reduced Expanded Disability Status Scale (EDSS) and recurrence rates, as well as improvements in quality of life [125]. Taken together, the pooled evidence suggests that most treated patients with MS improve or remain stable after MSC-based therapy [119]. Meta-analytic evidence specifically evaluating autologous MSC transplantation in MS has reported low transplant-related mortality and encouraging rates of no evidence of disease activity, with a greater benefit observed with intrathecal administration [126]. However, efficacy has not been definitively established in controlled studies, and long-term efficacy data remain preliminary [127]. Consistent with these observations, a recent systematic review and meta-analysis of randomized controlled trials, which included five randomized trials of MSC transplantation in MS, reported that MSC therapy did not significantly improve the number of lesions, lesion volume, or EDSS scores compared with control groups, though MSC treatment did not increase the incidence of adverse events; the authors emphasized that the small number of randomized trials available warrants further confirmation or revision of these findings [128]. Connick et al. administered autologous MSCs to patients with secondary progressive MS; the study reported improvements in visual function, reduced visual evoked response latency, and increased optic nerve area, with no serious adverse events. These structural and functional findings were consistent with a potential neuroprotective effect, although the uncontrolled, proof-of-concept design precludes definitive conclusions [129]. In a randomized, sham-controlled trial enrolling patients with active progressive MS, autologous MSC administration was well tolerated and significantly reduced the proportion of patients experiencing treatment failure, while a higher proportion of treated patients exhibited no evidence of disease activity, particularly with intrathecal administration; however, these short-term findings warrant confirmation in larger phase III trials [130]. 

Overall, the MS evidence remains mechanistically consistent but clinically inconclusive: preclinical data suggest anti-inflammatory, neuroprotective, and remyelinating activity, and uncontrolled human data are consistent with a potential neuroprotective effect, yet randomized evidence is limited and discordant, with pooled analyses showing no significant effect on lesion burden or EDSS and no increase in the incidence of adverse events, while the available sham-controlled trial reported reduced treatment failure, particularly with intrathecal administration. Surrogate signals such as visual evoked response latency and optic nerve area remain unlinked to clinically meaningful outcomes, and adequately powered phase III trials with standardized cell source, dose, and route of administration are still required.

T1D

T1D is a heterogeneous chronic disorder characterized by the autoimmune-mediated destruction of pancreatic β-cells [131]. T1D predominantly affects children and young individuals and accounts for approximately 5-10% of all diabetes cases [132]. The pathophysiology of T1D involves both humoral and cellular immunity. Humoral autoimmunity is characterized by the development of autoantibodies against self-antigens such as insulin, glutamic acid decarboxylase, and zinc transporter 8 [133]. Regarding cellular immunity, CD8+ cytotoxic T cells are the predominant immune cells recruited to pancreatic islets, while macrophages also play a crucial role in the pathological immune response in T1D [134]. B cells also contribute to T1D pathogenesis, with their antigen-presenting capacity representing an important pathogenic mechanism [135].

In preclinical models of T1D, MSC-based therapies have shown the potential to reverse disease progression, reduce inflammation, and suppress pathological immune responses [136]. ADSC treatment reduced pancreatic inflammation by attenuating the CD4+ Th1 immune response and promoting Treg expansion, thereby contributing to the maintenance of functional β-cells [137]. Preliminary findings showed that BM-MSCs exerted immunomodulatory effects by promoting immune tolerance in T1D [115]. Additionally, MSC-Exos have shown the ability to inhibit the development of Th1 and Th17 cells, leading to the restoration of the balance between Th1- and Th2-mediated immune responses in T1D [138].

Over the years, MSC-based therapies have gained increasing attention for the treatment of T1D due to their immunomodulatory properties and the release of bioactive products, including cytokines and EVs [139]. Across different clinical trials, MSC-based therapies showed a good safety profile, with no serious adverse events reported during treatment or follow-up [140]. A study conducted by Hu et al. showed a good safety profile of UC-MSCs in patients with new-onset T1D, with significant improvements in HbA1c and C-peptide levels, reduced insulin requirements, and no episodes of ketoacidosis during follow-up [141]. In a pilot randomized controlled open-label clinical study, Wu et al. followed patients with T1D who received UC-MSCs for eight years, reporting sustained safety during follow-up and a lower incidence of chronic diabetic complications in treated patients [142]. However, given the small sample size inherent to a pilot study and the open-label design, these long-term findings should be regarded as preliminary and warrant confirmation in larger, adequately powered controlled trials. A pilot randomized controlled open-label clinical study by Cai et al. evaluated the co-transplantation of UC-MSCs with autologous bone marrow cells in patients with established T1D, reporting an acceptable safety profile and moderate improvements in measures of insulin secretion [143]. More broadly, recent critical appraisals of cell-based therapy for T1D caution that, despite years of investigation, uncertainty persists and a definitive conclusion regarding efficacy cannot yet be drawn, reinforcing the need for adequately powered randomized trials [144]. Systematic overviews of stem cell-based clinical trials in diabetes mellitus similarly point to the limited number of completed, adequately powered randomized trials and the considerable heterogeneity in study protocols, underscoring the need for standardized evaluation [145]. In line with these observations, a meta-analysis of clinical trials concluded that MSC transplantation could represent a possible therapeutic option for diabetes, although additional well-designed randomized studies are required to confirm these findings [146].

Overall, the T1D evidence suggests biological plausibility and short-term safety but not established efficacy: preclinical models have shown the potential to reverse disease progression, reduce pancreatic inflammation, and preserve β-cell function, whereas human data derive mainly from small, open-label studies reporting reductions in HbA1c and insulin requirements, increased C-peptide, and, in a single pilot trial with long-term follow-up, a lower incidence of chronic diabetic complications. Randomized controlled evidence remains limited to randomized but open-label pilot trials with modest effects on insulin secretion, and the surrogate markers used - HbA1c, C-peptide, and insulin requirements - have not been linked to definitive clinically meaningful endpoints, so adequately powered, blinded controlled trials with standardized protocols are still required. 

SLE

SLE can be defined as a chronic inflammatory condition caused by an autoimmune response involving both the innate and adaptive immune systems, leading to damage in multiple organs and tissues, including the skin, kidneys, lungs, brain, blood vessels, and joints [147]. SLE is characterized by a dysregulated immune response that contributes to tissue injury through the production of inflammatory cytokines and aberrant T- and B-cell activation, with the subsequent production of autoantibodies that perpetuate damage across multiple organs [148]. A Th17/Treg imbalance is associated with the pathogenesis of SLE, contributing to an increased pro-inflammatory response [149]. Abnormal T-cell differentiation toward Th2 cells, together with their activation and associated cytokines, seems to play a vital role in the pathogenesis of SLE, ultimately contributing to B-cell hyperactivation [150].

Preclinical models of SLE have demonstrated the efficacy of MSC-based therapies in addressing lupus-associated complications such as lupus nephritis, with reductions in autoantibody levels and pro-inflammatory cytokines including IL-2, IL-12, IL-17, and IFN-γ [151]. In another study, MSCs showed recovery of Foxp3+ cells and downregulation of Th17 levels, accompanied by reductions in serum levels of anti-dsDNA antibodies and antinuclear antibodies (ANA) [152]. Preclinical results have also shown the therapeutic potential of MSC-EVs through their ability to inhibit pro-inflammatory responses and suppress autoimmune disease pathogenesis [153].

In patients with severe SLE refractory to conventional treatment, MSC-based therapies have been associated with clinical remission and improvement in organ dysfunction, together with an acceptable short-term safety profile in early-phase and frequently uncontrolled studies [154]. Clinical studies have reported lower proteinuria levels and Systemic Lupus Erythematosus Disease Activity Index (SLEDAI) scores in patients treated with MSC-based therapies compared with control groups [155]. Consistent with these observations, aggregate evidence restricted to randomized controlled trials, as synthesized in a recent systematic review and meta-analysis, indicates that MSC transplantation reduces disease activity and proteinuria relative to control interventions without increasing the incidence of adverse events; however, because only two randomized trials contributed to the disease-activity and renal analyses, these pooled estimates should be interpreted with caution and require confirmation in larger randomized trials [128]. In a small phase I clinical trial, allogeneic ADSCs administered to patients with refractory lupus nephritis were associated with reduced proteinuria and decreased disease activity, without serious adverse events; however, these findings should be regarded as preliminary evidence of therapeutic potential [156]. A case study by Liang et al. described the use of MSCs for diffuse alveolar hemorrhage, a rare but life-threatening complication of SLE, in a patient who did not respond to conventional pharmacological treatment; following MSC transplantation, the patient showed considerable clinical improvement, although single-case observations cannot establish therapeutic efficacy [157]. Taken together, however, clinical responses were not uniform, with some patients showing sustained improvement whereas others experienced relapse or no measurable response, and controlled studies with longer follow-up remain necessary [158].

Across the translational continuum, the SLE evidence remains dominated by preclinical and uncontrolled human observations. In preclinical models, MSC and MSC-EV administration consistently reduced autoantibody and pro-inflammatory cytokine levels, ameliorated lupus nephritis, and restored the Foxp3+/Th17 balance. In humans, early-phase uncontrolled studies reported clinical remission, improved organ function, and reductions in proteinuria and SLEDAI that must be interpreted cautiously given their uncontrolled designs. Randomized controlled evidence remains minimal, yet the pooled estimates restricted to randomized trials indicate reduced disease activity and proteinuria without an increased incidence of adverse events, although they rest on only two trials. Surrogate markers such as proteinuria, anti-dsDNA, and ANA have not been shown to translate into durable clinically meaningful benefit. The principal gaps are therefore the non-uniform clinical responses observed - with relapse or no measurable response in some patients - and the absence of adequately powered controlled trials with longer follow-up.

RA

RA is a chronic autoimmune disease affecting synovial joints, causing symptoms such as arthralgia, swelling, and limitation of movement, ultimately leading to articular cartilage destruction [159]. The interplay between the adaptive and innate immune systems plays a crucial role in RA pathogenesis. Abnormalities in the immune response lead to the production of autoantibodies, with rheumatoid factor (RF) being one of the most relevant [160]. T cells are also central players in RA pathogenesis, with an imbalance between TH1 and TH2 cells, TH17 overexpression, cytokine release by these cells, and impaired Treg function [161]. Synovial tissue in RA can be considered a tertiary lymphoid tissue, with a structure resembling secondary lymphoid tissues and containing sites for T- and B-cell differentiation. Within this tissue, abundant chemokines and pro-inflammatory factors promote inflammatory cell infiltration into the joints, contributing to pannus formation and synovial hyperplasia [162]. Fibroblast-like synoviocytes (FLSs) are specific fibroblasts located in the synovial membrane of the joints. Under the pro-inflammatory conditions present in RA, FLSs exhibit increased proliferation, reduced apoptosis, and increased expression of cytokines and chemokines, contributing to pannus formation [163].

Preclinical models of MSC-based therapies for RA have shown that MSCs can reduce the incidence and severity of the disease [136]. MSCs could immunomodulate the inflammatory response by inhibiting T-cell responses and promoting Treg cell induction, thereby contributing to decreased disease severity [164]. A study conducted by Liu et al. showed that MSCs inhibited the proliferation, invasive behavior, and inflammatory responses of FLSs, accompanied by reduced disease severity in a preclinical model of RA [165]. MSC-Exos exhibit therapeutic potential due to their ability to modulate immune responses, reduce pro-inflammatory cytokine production, and decrease synovial inflammation [166].

Clinical trials of MSC-based therapies for RA have generally shown an acceptable short-term safety profile in early-phase studies, with no serious adverse events reported [167]. However, these findings derive largely from small, heterogeneous, and frequently uncontrolled studies, and the efficacy of MSC-based therapy in RA has not been definitively established [168,169]. In 2010, Ra et al. started the first clinical study of MSC-based therapy for RA, reporting a favorable short-term safety profile and clinical improvements, including reductions in Visual Analog Scale (VAS) and Korean Western Ontario and McMaster Universities Osteoarthritis Index (KWOMAC) scores and discontinuation of corticosteroid therapy in two patients; as an early uncontrolled experience, however, these findings should be regarded as preliminary [114]. In a randomized, triple-blind, placebo-controlled phase 1/2 clinical trial, intra-articular administration of autologous BM-MSCs in RA patients with knee involvement showed improvements in pain and functional outcomes, including Western Ontario and McMaster Universities Osteoarthritis Index (WOMAC) scores and standing time, together with reductions in methotrexate and prednisolone use; however, most between-group differences did not reach statistical significance, warranting cautious interpretation [170]. Ghoryani et al. evaluated immunological, clinical, and laboratory outcomes in RA patients treated with autologous MSCs, reporting a decrease in peripheral Th17 cells and an increase in Tregs, together with reductions in DAS28-erythrocyte sedimentation rate (ESR) and VAS scores, RF levels, and overall disease severity; although these findings suggest immunomodulatory activity, they derive from a single study and require confirmation in larger controlled trials [171].

The RA evidence remains inconclusive despite the availability of a randomized, placebo-controlled trial: preclinical data show reduced disease severity, T-cell suppression, Treg induction, and inhibition of FLS proliferation and synovial inflammation, while human evidence derives largely from small, heterogeneous, often uncontrolled studies reporting reductions in DAS28-ESR, VAS, and RF with corticosteroid and methotrexate sparing. The single randomized, triple-blind, placebo-controlled trial of intra-articular BM-MSCs improved pain, WOMAC scores, and standing time, but most between-group differences were not statistically significant, and because surrogate markers such as DAS28-ESR and RF remain unlinked to durable clinically meaningful outcomes and the immunological improvements in refractory RA come from a single uncontrolled study, adequately powered controlled trials with standardized cell source, dose, and route are still required.

Current limitations, challenges, and future perspectives of MSC-based therapies

MSC-based therapies have shown therapeutic potential for autoimmune diseases, with promising results across preclinical and clinical studies (Table 1) aimed at translating the biological and immunomodulatory properties of MSCs into clinical applications, generally showing an acceptable short-term safety profile in early-phase and frequently uncontrolled studies. However, given the limited follow-up and methodological heterogeneity of these studies, the absence of serious adverse events should not be interpreted as definitive evidence of long-term safety. Nevertheless, although MSC-based therapies could represent a viable treatment for autoimmune diseases, their efficacy remains inconsistent across studies, potentially due to variations in MSC sources, cultivation techniques, and treatment protocols [125]. Additionally, key considerations for MSC-based therapies include MSC dosage, route of administration, treatment duration, and the immune status of the recipient, among others [47]. Furthermore, the lack of uniformity in MSC characterization represents an important limitation, highlighting the need for updated characterization guidelines and improved standardization to better account for MSC variability and enable more reliable comparisons across studies [172]. In this context, Stroncek et al. demonstrated functional and molecular differences among MSCs produced by different laboratories even when the same bone marrow starting material was used, with inter-laboratory manufacturing differences contributing more to MSC variability than the source material itself [173]. To address this limitation, the recent Delphi-driven consensus on MSC definition and clinical trial reporting aims to move beyond the 2006 minimal criteria, providing recommendations that address product heterogeneity and improve transparency and reproducibility in MSC-based clinical research [174]. Specific protocols should also be developed to optimize MSC production and survival following administration [175].

**Table 1:** Summary of preclinical evidence and reported clinical outcomes of MSC- and MSC-EV-based therapies in selected autoimmune diseases MS: Multiple sclerosis; T1D: Type 1 diabetes mellitus; SLE: Systemic lupus erythematosus; RA: Rheumatoid arthritis; MSC: Mesenchymal stem cell; MSC-EV: Mesenchymal stem cell-extracellular vesicle; EDSS: Expanded Disability Status Scale; MSC-Exos: Mesenchymal stem cell-derived exosomes; UC-MSC: Umbilical cord-derived mesenchymal stem cell; SLEDAI: Systemic Lupus Erythematosus Disease Activity Index; FLS: Fibroblast-like synoviocyte; ESR: Erythrocyte sedimentation rate; VAS: Visual Analog Scale; RF: Rheumatoid factor; WOMAC: Western Ontario and McMaster Universities Osteoarthritis Index; BM-MSC: Bone marrow-derived mesenchymal stem cell

Disease	Key Pathophysiological Features	Preclinical Evidence	Reported Clinical Outcomes	Evidence Source/Design	Evidence Limitations
MS	Demyelination and neuroaxonal degeneration involving autoreactive immune cells, microglial activation, oxidative stress, and mitochondrial dysfunction [116-118]	MSCs reduced inflammation, preserved axons, promoted oligodendrogenesis and remyelination, and improved nerve conduction [120,121]. MSC-EVs showed neuroprotective and neuroregenerative effects [122].	↓ Recurrence rates and EDSS; improved quality of life [124,125]. Improved visual function, ↓ visual evoked response latency, and ↑ optic nerve area were also reported [129]; favorable short-term safety profile [124,125,129].	Systematic review of MSC therapy in MS [124]; systematic review and meta-analysis of randomized controlled trials [125]. Open-label, uncontrolled phase 2a proof-of-concept study [129]. Systematic review and meta-analysis of randomized controlled trials of MSC transplantation in MS, including five randomized trials [128].	Small samples, open-label and uncontrolled designs, and heterogeneity in MSC source, dose, and route of administration, with limited long-term efficacy data [124,125,129]. The pooled randomized evidence derives from a small number of trials and showed no significant improvement in the number of lesions, lesion volume, or EDSS scores, though it did not indicate an increased incidence of adverse events [128].
T1D	Pancreatic β-cell destruction involves humoral and cellular immunity, with autoantibody production and participation of CD8+ T cells, macrophages, and B cells [131-135]	MSCs reduced pancreatic inflammation and pathological immune responses, promoted Treg expansion and immune tolerance, and contributed to β-cell preservation [115,136,137]. MSC-Exos inhibited Th1/Th17 responses and restored Th1/Th2 balance [138].	↓ HbA1c and insulin requirements; ↑ C-peptide levels; with no serious adverse events [140,141]. Long-term follow-up suggested sustained safety and a lower incidence of chronic T1D complications [142].	Systematic review and meta-analysis of randomized controlled trials of MSC-based therapy in T1D [140]; clinical study of Wharton's jelly/UC-MSC implantation in newly onset T1D [141]; pilot randomized controlled open-label clinical study with 8-year follow-up, in which UC-MSCs were co-transplanted with autologous bone marrow cells [142]	Small sample sizes, absence of blinding and placebo control, and heterogeneity in MSC source, dose, and protocol; larger, adequately powered controlled trials are required. The long-term follow-up data derive from a single pilot, open-label study and remain preliminary [140-142].
SLE	Multiorgan inflammatory disease characterized by dysregulated innate and adaptive immunity, aberrant T- and B-cell activation, autoantibody production, and Th17/Treg imbalance [147-150]	MSCs ameliorated lupus nephritis, reduced autoantibody and pro-inflammatory cytokine levels, restored Foxp3+ cells, and downregulated Th17 responses [151,152]. MSC-EVs inhibited pro-inflammatory responses and autoimmune disease progression [153].	↓ Proteinuria and SLEDAI scores; clinical remission and improvement in organ dysfunction in severe/refractory SLE; favorable short-term safety profile [154-156]. Clinical improvement was also reported in refractory diffuse alveolar hemorrhage [157].	Uncontrolled four-year experience of allogeneic MSC transplantation in severe and refractory SLE [154]; aggregate assessment of efficacy and safety [155]; phase I clinical trial in refractory lupus nephritis [156]; single-case report of diffuse alveolar hemorrhage [157]. Systematic review and meta-analysis of randomized controlled trials with disease-specific pooled analyses of SLEDAI and proteinuria, based on two randomized trials [128].	Findings derive predominantly from early-phase and frequently uncontrolled studies, together with a single-case observation; clinical responses were not uniform, with relapse or no measurable response in some patients, and controlled studies with longer follow-up remain necessary [154-157]. The pooled disease-activity and renal estimates rest on only two randomized trials and require confirmation in larger randomized trials [128].
RA	Synovial inflammation characterized by immune dysregulation, autoantibody production, Th17/Treg imbalance, FLS hyperactivation, pannus formation, and progressive cartilage destruction [159-163]	MSCs reduced disease severity, inhibited T-cell responses, promoted Treg induction, and suppressed FLS proliferation and inflammatory activity [136,164,165]. MSC-Exos reduced pro-inflammatory cytokine production and synovial inflammation [166].	↓ DAS28-ESR, VAS, and RF levels; pain and functional outcomes improved, including WOMAC scores; corticosteroid and methotrexate use decreased, with a favorable short-term safety profile [167,170,171].	Narrative review of MSC-based therapy in RA [167]; randomized, triple-blind, placebo-controlled phase 1/2 clinical trial of intra-articular autologous BM-MSCs [170]; uncontrolled clinical study in refractory RA [171]	Findings derive largely from small, heterogeneous, and frequently uncontrolled studies, and the efficacy of MSC-based therapy in RA has not been definitively established [168,169]; most between-group differences in the randomized trial did not reach statistical significance [170]; the immunological and clinical improvements reported in refractory RA derive from a single study and require confirmation in larger controlled trials [171]

Another critical consideration concerns the immunogenicity of allogeneic MSCs. Although traditionally regarded as hypoimmunogenic or "immune privileged," MSCs are now more accurately described as "immune evasive, not immune privileged" [176]. Indeed, allogeneic MSCs remain susceptible to immune attack by the recipient, and their immunogenicity can be enhanced under pro-inflammatory conditions, potentially triggering allogeneic immune reactions that compromise long-term efficacy [177]. This is especially relevant for the chronic treatment of autoimmune diseases, in which repeated infusions are commonly required, and the heterogeneity of MSC-based products may further complicate the prediction of such immune responses, which could support the consideration of monitoring anti-HLA and donor-specific antibodies in clinical protocols [178].

Beyond the biological and manufacturing limitations discussed above, the clinical translation of MSC-based therapies has been further complicated by the commercialization of unproven stem cell-based interventions, a global problem that has proven resistant to regulatory efforts and has prompted calls for coordinated national and international responses [179]. These observations reinforce the need for strengthened regulatory vigilance and for conducting MSC-based research and therapy within the framework of controlled clinical trials.

Clinical evidence for MSC-EV- and MSC-Exo-based therapies remains limited and predominantly confined to the preclinical stage. However, several challenges remain in translating these promising preclinical findings into clinical applications, including scalable production, controlled biodistribution, release kinetics, cargo optimization, and a better understanding of their mechanisms of action [180]. Moreover, the clinical application of MSC-Exos faces additional challenges related to the lack of standardized isolation and purification protocols, batch-to-batch variability, storage conditions, and biosafety [181]. These barriers are now being addressed by dedicated consensus and regulatory initiatives, which have emphasized the need for harmonized quality and regulatory frameworks, standardized analytical approaches, and coordinated collaboration among regulatory authorities, industry, and academic investigators in order to accelerate the responsible clinical development of EV-based products [182,183]. In addition, in line with the MISEV2023 guidelines, rigorous functional characterization of EVs and the clear distinction between vesicle-mediated effects and those elicited by co-isolated soluble factors are required, given that attributing therapeutic activity to EVs alone demands appropriate control experiments [184].

Future research should therefore focus on overcoming these limitations and optimizing the translation of MSC- and MSC-EV-based therapies into more consistent, personalized, and clinically applicable therapeutic strategies. In this context, subsequent therapeutic approaches should also consider the potential influence of concomitant pharmacological treatments on MSC activity and clinical outcomes. Illustrating this complexity, a randomized study evaluating MSCs in combination with corticosteroids for acute GVHD was terminated early due to futility, raising the possibility that concomitant immunosuppressive treatments may influence MSC-mediated immunomodulation [185]. This experience highlights the importance of considering concomitant pharmacological treatments and other clinical parameters in these MSC-based therapeutic approaches, integrating MSCs within broader and longitudinal treatment strategies. The integration of stem cell-based therapies into personalized medicine represents an attractive perspective, aiming to tailor therapeutic strategies according to individual patient characteristics [186]. Accordingly, MSCs could be further explored not only as stand-alone therapies, but also as components of broader, multimodal, and individualized therapeutic strategies. 

The progression of exosome-based therapies toward clinical translation has been encouraging, and further advances in this field could facilitate their incorporation into clinical practice [50].

## Conclusions

MSCs and their secretome, particularly MSC-EVs and exosomes, represent potential therapeutic tools and promising investigational therapeutic approaches for immune-mediated disorders. Their immunomodulatory capacity-exerted through the regulation of innate and adaptive immune cell populations, modulation of pro- and anti-inflammatory cytokines, downregulation of pro-inflammatory responses, including Th1/Th17 responses, and promotion of regulatory phenotypes such as Tregs, Bregs, and M2 macrophages-provides a multifaceted biological mechanism capable of regulating inflammatory responses and promoting immune homeostasis. These properties have generated increasing interest in the potential application of MSC-based therapies for autoimmune diseases.

Preclinical and clinical evidence across MS, T1D, SLE, and RA supports the therapeutic potential of MSC- and MSC-EV-based interventions, together with an acceptable short-term safety profile in early-phase and frequently uncontrolled studies. Nevertheless, the absence of serious adverse events in these studies should not be interpreted as definitive evidence of long-term safety, and the available efficacy data should be regarded as preliminary evidence of therapeutic potential rather than established efficacy. Clinical studies have reported improvements across disease activity scores, biomarker profiles, and disease-specific functional outcomes; however, clinical translation remains constrained by manufacturing heterogeneity, variability in MSC dosage, route of administration, and treatment duration, as well as the need for standardized characterization, isolation, and production protocols.

The future of MSC-mediated immunomodulation will depend on overcoming these translational barriers while further exploring the integration of MSC-based therapies into multimodal and personalized treatment strategies. Understanding the crosstalk between MSCs and the host inflammatory microenvironment, including the potential influence of concomitant pharmacological therapies, may be critical for optimizing their therapeutic application. Integrating cell- and EV-based therapies within longitudinal and patient-tailored therapeutic frameworks positions MSC-mediated immunomodulation as a promising field for the continued development of regenerative and personalized medicine. 
